# Protocol for translation and cross-cultural adaptation of diagnostic questionnaires for pediatric disorders of gut-brain interaction

**DOI:** 10.1590/1984-0462/2025/43/2024191

**Published:** 2025-03-24

**Authors:** Thaís Moreno Tomé, Ana Beatriz de Menezes Lima, Janaína Mezzonato Machado, Mariana Tschoepke Aires, Silvio da Rocha Carvalho, José Cesar da Fonseca Junqueira, Carlos Fernando Francesconi

**Affiliations:** a Universidade Federal do Rio de Janeiro, Instituto de Puericultura e Pediatria Martagão Gesteira, Rio de Janeiro, RJ, Brazil.; b Universidade Federal do Rio Grande do Sul, Hospital de Clínicas de Porto Alegre, Porto Alegre, RS, Brazil.

**Keywords:** Cross-cultural comparison, Surveys and questionnaires, Clinical diagnosis, Brain-gut axis; Pediatrics, Comparação transcultural, Inquéritos e questionários, Diagnóstico clínico, Eixo encéfalo-intestino; Pediatria

## Abstract

**Objective:**

To describe the protocol used for translation and cross-cultural adaptation of the questionnaires developed by the Rome Foundation for the diagnosis of disorders of gut-brain interaction in the pediatric population.

**Methods:**

The protocol was proposed based on a narrative review of the literature on the cultural adaptation process of measurement instruments in epidemiology, analyzing its stages, and verifying its use and feasibility. The guidelines for the cross-cultural adaptation of diagnostic instruments developed by the Rome Foundation, which defines and periodically reviews diagnostic criteria, were incorporated into the protocol.

**Results:**

The proposed protocol includes: (i) preparation; (ii) forward translation; (iii) reconciliation; (iv) backward translation; (v) review of the backward translation; (vi) cognitive debriefing; (vii) final review; (viii) calculation of the item content validity index; and (ix) approval by the Rome Foundation.

**Conclusions:**

The methodological steps described in this protocol may contribute to future translations and cross-cultural adaptations of diagnostic questionnaires of disorders of gut-brain interaction and other materials from the Rome Foundation, enabling their use in epidemiological studies.

## RESUMO

**Objetivo:**

Descrever o protocolo utilizado para tradução e adaptação transcultural dos questionários elaborados pela Fundação Roma para o diagnóstico de distúrbios da interação intestino-cérebro na população pediátrica.

**Métodos:**

O protocolo foi proposto com base em revisão narrativa da literatura sobre processo de adaptação cultural de instrumentos de aferição em epidemiologia, analisando suas etapas, verificando seu emprego e exequibilidade. Foram incorporadas ao protocolo, as diretrizes para a adaptação transcultural dos instrumentos diagnósticos elaborados pela Fundação Roma, que define e revisa periodicamente os critérios diagnósticos.

**Resultados:**

O protocolo proposto inclui: (i) preparação; (ii) tradução; (iii) reconciliação; (iv) retrotradução; (v) revisão da retrotradução; (vi) *debriefing* cognitivo; (vii) revisão final; (viii) cálculo do índice de validade de conteúdo; e (ix) aprovação pela Fundação Roma.

**Conclusões:**

Os passos metodológicos descritos neste protocolo poderão contribuir para futuras traduções e adaptações transculturais de questionários diagnósticos de distúrbios da interação intestino-cérebro e demais materiais da Fundação Roma, possibilitando seu emprego em estudos epidemiológicos.

## INTRODUCTION

Disorders of gut-brain interaction (DGBIs), previously known as functional gastrointestinal disorders, comprise a variety of chronic or recurrent symptoms not explained by structural or biochemical abnormalities and not attributed to any other condition after proper medical evaluation.^1,2,3,4,5^ Symptoms result from a complex interaction of factors such as intestinal dysbiosis, altered mucosal immune function, visceral hypersensitivity, and dysregulation of intestinal signaling and motor function by the central, peripheral, and enteric nervous system.^4^

Although very common, with a prevalence of 27% to 40.5% in children up to 3 years of age and from 9.9% to 27.5% in older children and adolescents, only in the last decades have the DGBIs been widely studied, categorized, and diagnosed.^3,4,5,6^ This is largely due to a group of gastroenterology experts who have compiled diagnostic criteria for these disorders in a consensus form, updated periodically, with the latest version published in 2016 (Rome IV Criteria).^3,7^ The Rome Foundation (RF) is an independent, non-profit organization dedicated to supporting science and education on the diagnosis and treatment of DGBIs, aiming to increase knowledge and improve patients’ quality of life.^8^

One major challenge in diagnosing and managing DGBIs is the lack of objective biochemical markers or structural abnormalities for diagnosis and inclusion criteria in studies.^1,2,4^ To address this, RF experts developed questionnaires that convert symptom-based criteria into patient-friendly questions, facilitating diagnosis.^9^ Three questionnaires were produced for pediatric patients, targeting different DGBIs and covering various sections related to symptoms: “Parent-Report Form for Neonates and Toddlers (0–3 years of age)”, “Parent-Report Form for Children and Adolescents (4 years of age and older)”, and “Self-Report Form for Children and Adolescents (10 years of age and older)”. The questionnaires comprise several sections and include items on the presenting symptoms, their location, frequency, duration, and intensity, targeting the different DGBIs (Table 1).

**Table 1.** Childhood and adolescent disorders of gut-brain interaction as defined by the Rome Foundation.^1,2^

Neonate/toddler	Child/adolescent
Cyclic vomiting syndrome	Abdominal migraine
Functional constipation	Aerophagia
Functional diarrhea	Cyclic vomiting syndrome
Infant colic	Functional abdominal pain-not otherwise specified
Infant dyschezia	Functional constipation
Infant regurgitation	Functional dyspepsia
Rumination syndrome	Functional nausea
	Functional vomiting
	Irritable bowel syndrome
	Nonretentive fecal incontinence
	Rumination syndrome

To ensure global use, the RF has initiated the “Rome Translation Project”, supervising the translation and cross-cultural adaptation of RF materials for use in local and global epidemiological and clinical studies.^10,11,12,13,14^ This complex process involves semantic, language, and cultural evaluation to maintain the original meaning and intention of the instruments in the adapted culture.^10,15^ While the diagnostic questionnaire for adults has been culturally adapted to over 25 languages, the pediatric questionnaires are currently undergoing the same process.^12,13,16^

The aim of this study was to propose a protocol for the translation and cross-cultural adaptation of the RF pediatric questionnaires for the diagnosis of DGBIs into Brazilian Portuguese. This will contribute to the availability of easy, practical, and valid tools for correct diagnosis, enabling reliable and reproducible studies in epidemiology, physiopathology, and treatment in children.

## METHOD

A narrative literature review was conducted to search for studies that performed the translation and/or cross-cultural adaptation of the RF pediatric questionnaires. The research strategy included the combination of the keywords: (*Rome questionnaire* OR *Rome criteria*) AND (*disorders of gut-brain interaction* OR *functional gastrointestinal disorders* OR *brain-gut axis*) AND (*cross-cultural translation* OR c*ultural adaptation* OR *transcultural adaptation* OR *cross-cultural comparison)* AND (*child* OR *children* OR *pediatrics* OR *neonates* OR *adolescents*) in the United States National Library of Medicine (PubMed), Scientific Electronic Library Online (SciELO), and Latin American and Caribbean Health Sciences Literature (Lilacs) databases. The search period was January 2000 to December 2023 and the filtered languages were English, Spanish, and Portuguese. Additionally, the coordinators of the RF translation project were consulted to verify if there was any cross-cultural adaptation of the pediatric questionnaires into Brazilian Portuguese.

Different protocols can be used for the translation and cross-cultural adaptation of instruments, each with its advantages and limitations. This study followed the universalist approach, and the methodological steps initially proposed by Herdman et al.,^17^ with adaptation and operational systematics proposed by Reichenheim and Moraes.^15^ It is a widely used protocol in cross-cultural adaptation studies involving sequential stages. In addition, RF guidelines were strictly followed.^18^

Herdman et al. proposed a method to maintain an instrument’s validity and reliability across cultures, involving the assessment of six types of equivalence: conceptual, item, semantic, operational, measurement, and functional.^17^ In 2007, Reichenheim and Moraes presented a detailed framework for the operationalization of cross-cultural adaptation, including the stages: translation and backward translation, expert committee review, pre-testing with cognitive interviews, and psychometric validation.^15^ Similarly, the RF guidelines outline the following steps: forward translation, reconciliation, backward translation, review of the backward translation, cognitive debriefing, final review, and approval by the foundation.^18^

This study was approved by the Research Ethics Committee of the Institute of Childcare and Pediatrics Martagão Gesteira (*Instituto de Puericultura e Pediatria Martagão Gesteira*) at the Federal University of Rio de Janeiro (UFRJ), under CAAE 45206621.8.0000.5264.

## RESULTS

### Narrative review of literature

In the SciELO and Lilacs databases, no study was found. In PubMed, seven results appeared: one compendium of abstracts not related to the topics; two that were related to DGBIs and mentioned Rome criteria but did not perform a cross-cultural translation; two that mentioned diseases related to the gut-brain axis but not DGBIs; one that performed a cross-cultural translation of another instrument; and one that was referring to the RF global epidemiology study in adults.

Although not indexed in these databases, the RF indicated an article published in 2019 describing the cross-cultural translation process of RF pediatric diagnostic questionnaires into the Spanish language.^19^ This study, carried out by the Latin American Society for Pediatric Gastroenterology, Hepatology and Nutrition (LASPGHAN, *Sociedad Latinoamericana de Gastroenterología, Hepatología y Nutrición Pediatrica*) after authorization from the RF, describes the validation and reproducibility of the Rome IV Criteria questionnaires translated into Spanish.^19^

### Protocol for the translation and cross-cultural adaptation of Rome Foundation questionnaires

For the translation of any RF questionnaires, its guidelines must be strictly followed, and prior authorization is required.^10^ The RF requests a research proposal with the justification for obtaining a version in the target language for which the instrument is intended to be translated, the objectives of its use, and the thorough methodology to be adopted. Once the proposal is approved, the foundation provides the questionnaires.

The RF oversees every stage of the project, appointing a direct point of contact and a clinical monitor who is a native speaker of the country where the cross-cultural adaptation will take place.^18^

The clinical monitor must be a physician, usually a gastroenterologist, with the following duties:

1. Approve the translators involved in the project and ensure that they comply with the Foundation’s guidelines;
2. Monitor the stages of forward translations, reconciliation, and backward translation; and
3. Ensure the highest quality possible.^20^

Once the process is completed, the translated and adapted version will be available from the RF at no cost to researchers conducting independent studies, making it possible to include the targeted culture in the Foundation’s global epidemiology studies.^10^

The steps defined for the cross-cultural adaptation of the questionnaires, and described below, are:

1. Preparation;
2. Forward translation;
3. Reconciliation;
4. Backward translation;
5. Backward translation review;
6. Cognitive debriefing;
7. Final review;
8. Calculation of the item content validity index; and
9. Approval by the Rome Foundation.

### 1. Preparation

At this stage, a literature review should be conducted to determine if there are any existing cross-cultural adaptations of RF questionnaires for the pediatric population. If no validated instrument is found, the next step is to evaluate the conceptual and item equivalence of the questionnaire. This involves examining the relevance of each domain of the original instrument in the targeted population and its ability to reflect the intended concept being investigated.^15,17,21^ To assess these types of equivalence, a committee of experts should be formed, consisting of pediatric gastroenterologists (three to five professionals) and other healthcare professionals (one or two), such as nutritionists, nurses, and physiotherapists related to the subject. This committee is responsible for analyzing the relevance of each item in the instrument, discussing its meaning and applicability. A comprehensive understanding of DGBIs, the latest Rome criteria, and their variations based on age range and symptom groups is crucial at this stage. It is also important to evaluate the response options in detail, as the Rome criteria often require conditional questions.

Once this stage has been completed and the absence of questionnaires adapted to the proposed sociocultural context has been established, formal contact with the RF should be started through the organization’s electronic portal. If the proposal is accepted, the RF officiates in a contract, containing the conditions of copyright and confidentiality, and the concession for the instruments’’ translation.

### 2. Forward translation

Two forward translations are recommended, made independently by two native translators of the targeted culture and recorded in writing.^22^ According to the RF guidelines, these translators must be professionals, fluent in English, and experienced in translating medical materials.^18^ This step generates two forward translations, called T1 and T2.

**Figure 1.**
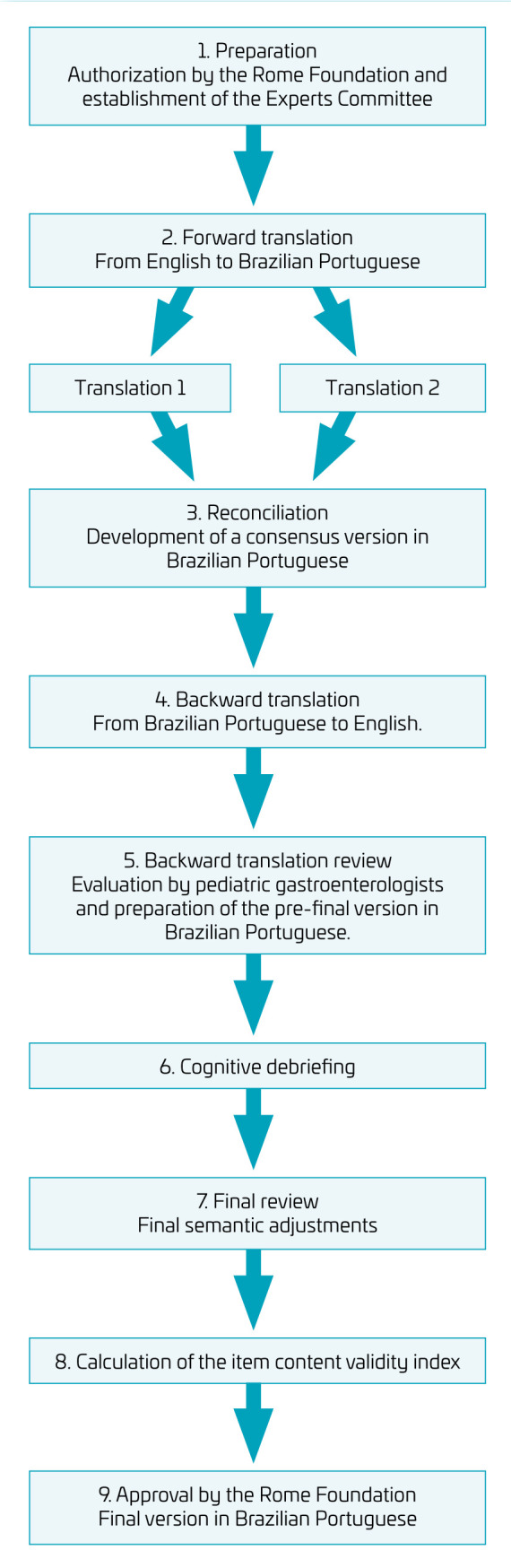
Flowchart of the cross-cultural translation process.

In the case of this protocol, translators should be aware of the research’s objectives and that the translation should not only be literal but also adapted to culturally accepted and relevant expressions in Brazil. Figure 1 shows the flowchart of the cross-cultural adaptation process.

### 3. Reconciliation

In this step, both forward translations (T1 and T2) must be combined into a single version, linguistically and semantically equivalent to the original to ensure the transfer of meaning between the different languages and provide a similar effect on the two cultures’ respondents.^15,22^ The same expert committee involved in the preparation step should meet with the clinical monitor of the RF to evaluate both translations, identify their differences, and perform a reconciliation process. It is appropriate to record the content of discussions and decisions for further consultation.

Brazil is a multicultural society, characterized by ethnic, cultural, and linguistic diversity. One aspect of cultural plurality is the use of colloquialisms. Colloquial language varies significantly depending on geographic location, local customs, and the inhabitants’ cultural background. Therefore, the expert committee should include specialists from different regions to ensure diverse language perspectives and the inclusion of a greater number of different expressions.

To achieve instrument equivalence, the expert committee carefully assesses all items based on the following concepts:^21,23^

- Semantic equivalence: includes evaluating vocabulary and grammar to ensure that the meaning is accurately transferred between languages.
- Idiomatic equivalence: as colloquialisms can be challenging to translate, the committee may need to propose equivalent expressions to maintain the intended meaning.
- Equivalence of experience: the items in the questionnaire should capture and evaluate the experiences of daily life in the local culture. If necessary, items can be replaced with similar ones commonly used in the target language’s culture.
- Conceptual equivalence: evaluation that the concept is the same in both versions, promoting the questionnaire’s acceptability and avoiding words that can be considered aggressive or taboo in the culture for which the instrument is being adapted.

The product of this step is a reconciled version to be backward translated.

### 4. Backward translation

This step, performed for quality control, aims to verify that the meaning of the original questionnaire was maintained during the forward translation and reconciliation processes.^22^ Therefore, a professional translator, with experience in medical materials, with English as native language, and fluent in Portuguese, carries out the backward translation of the reconciliation version from Brazilian Portuguese into English. Complying with the protocols of the RF as well as the recommendation of other authors, the translator should not be aware of the original version nor be involved in the initial forward translation.^18,22^

### 5. Backward translation

To increase the rigor of the process, the backward translation is evaluated by three other pediatric gastroenterologists (one for each questionnaire) not previously involved in the process, comparing the original questionnaire and the backward translation.

They evaluate each item and instruction in:

1. Unchanged;
2. Minor changes;
3. Major changes, or
4. Completely changed, with a field to add their observations and suggestions.

The Expert Committee reviews the experts’ assessment and decides which changes must be made and incorporated into the pre-final version of the instrument, to be used in the next step.

### 6. Cognitive debriefing

The cognitive debriefing is a relevant step in evaluating the interviewees’ understanding of the questionnaire and the acceptability of the instrument’s format.^24^ This should be performed by the main investigator, after appropriate training for a cognitive semi-structured face to face interview. It is essential that the investigator encourages respondents to express their thoughts, feelings, and suggestions aloud when reading each item of the instrument.^22^ Interviews should be conducted with the targeted population, excluding individuals with any comorbidity, and temporary or permanent disability.^24^

The cognitive debriefing procedure is based on the recommendations of the RF, which determines the number of cognitive interviews: two parents/caregivers or adolescents (according to the age to which each questionnaire refers) should be interviewed separately and in person.^18^ The pre-final version of the questionnaire is printed and reviewed to ensure it is clear, understandable, and meets acceptance standards. The informed consent form is read and signed by parents or legal guardians and the audio of the interview is recorded for further analysis. It is important that the informed consent form states clearly how long the audio will be stored before it is discarded or, if it is not discarded, that anonymity is warranted. For each item of the questionnaire, instructions and answers, the investigator must ask the following questions: “did you understand what is written?” (possible answers: “fully understood”; “only understood one part”; “did not understand anything”; and “I cannot say”), “could you rephrase the question?” and “would you make any modifications to improve understanding?”. If so, respondents should be encouraged to propose amendments to make the content clearer and more understandable. The procedure is repeated until it reaches more than 90% acceptance and understanding of the questionnaire. The participants’ suggestions during the cognitive interviews must be incorporated to produce a draft version to be proposed to the experts’ committee by the main investigator.

### 7. Final review

The experts’ committee evaluates the cognitive debriefing, discusses, and decides on possible changes to be implemented to obtain the final version of the questionnaire.

### 8. Calculation of the item content validity index

The item content validity index (ICVI) is a quantitative assessment that evaluates whether the content of the instrument is appropriate and relevant for the target culture.^25^ It is widely used in studies of cross-cultural translation and examines the agreement between evaluators regarding the instrument and its items systematically, ensuring that the instrument not only maintains its original validity but is also culturally sensitive and appropriate for the target population. Using a Likert-like scale with 1–4 scores, each instruction, question, and answer are individually appreciated, allowing an initial assessment of its clarity and relevance for the target population.^25^

A team of five to seven judges, physicians specialized in pediatric gastroenterology who did not participate in any of the previous stages, complete an evaluation form of each item, considering its importance and conceptual equivalence to the original instrument. At this stage, it is also advisable that the judges involved be native from the various regions of Brazil.

For each component of the questionnaire, the judges report their responses in four scores:

1. Item not equivalent;
2. Item requires major changes;
3. Item requires minor changes; and
4. Item totally equivalent.

The ICVI score is calculated by adding the concordances for items marked as “3” or “4” by the judges and calculating their proportions. Items with scores of “1” or “2” are usually subject to review or exclusion. A higher ICVI indicates a higher level of agreement among experts regarding the validity of the instrument’s content after the cross-cultural translation.^25^

### 9. Approval by the Rome Foundation

Finally, the final version of the instrument is submitted for approval by the RF, along with the clinical monitor’s recommendation letter, the two initial forward translations, the backward translation, the version used for cognitive debriefing, and the comments made by the respondents.

## DISCUSSION

In Brazil, studies on the prevalence of DGBIs using Rome criteria in the pediatric population are lacking. An important first step to filling this gap is the availability of validated instruments capable of detecting those disorders in large epidemiologic studies. This is the first study to systematize a protocol for translation and cultural adaptation of the questionnaires for the diagnosis of pediatric DGBIs, aiming to provide validated instruments for accurate diagnosis and optimize the initiation of treatment.

This protocol was based on Reichenhein and Herdman’s guidelines, worldwide references on the subject.^15,17^ The process of cross-cultural translation is less time-consuming and costly than developing new instruments. Using equivalent questionnaires allows for standardization in scientific research and comparability between national and international studies, providing a reliable tool for epidemiological research and contributing to relevant information for the definition and evaluation of public policies.

For DGBIs, the only official and available tools for epidemiological study are those developed by the RF, based on Rome Criteria, which are constantly revised and improved based on validation studies.^26^

It is important to highlight the difficulty in conducting factor analysis for RF questionnaires, as categorical variables are not eligible for this type of evaluation, and the instrument contains both dichotomous and Likert scale questions.^27^ Due to the presence of approximately 30% categorical variables in the pediatric RF questionnaires, factor analysis was not included in the psychometric evaluation protocol of these instruments.

This study should be considered in light of certain limitations. The RF guidelines recommend that cognitive debriefing be performed with two families or adolescents until 90% acceptance and understanding of the questionnaire. If acceptance is reached in the first or second round of interviews, the minimum number of 5 to 40 individuals recommended by certain authors will not be adopted.^22,24^ Also, the RF guidelines focus on the translation process and do not incorporate the analysis of psychometric properties. Regarding validity, we recommend that experts from all five Brazilian regions evaluate the content validity of each item, considering the cultural and language differences.

In conclusion, we present the minimum requirements requested by the RF in this protocol, which can be adapted to include a larger number of individuals and the subsequent performance of psychometric tests. Finally, we expect that this publication will contribute to systematizing and facilitating the process of translation and cross-cultural adaptation of current and future RF instruments.

## Data Availability

The database that originated the article is available with the corresponding author.
